# Pros and cons of student journals

**DOI:** 10.1007/s40037-015-0244-2

**Published:** 2016-01-11

**Authors:** Omar Ali Aboshady, Mohamed Alaa Gouda

**Affiliations:** 1 Faculty of Medicine, Menoufia University, Shebin El-Kom, Menoufia, Egypt; 2 Department of Oncology, Faculty of Medicine, Menoufia University, Menoufia, Egypt

Student participation in research has become an important practice for medical students to address the growing emphasis on evidence-based medicine [1]. Student journals are student-led peer-reviewed journals, which provide platforms dedicated to the publishing of student research. These journals are usually started by interested students (supported by faculty staff) in universities, national student associations or independent groups.

Over the past few years, there has been a rapid increase in the number of these journals, which include the Journal of Young Investigators, International Journal of Medical Students, Res Medica Journal, McGill Journal of Medicine, and Asian Students’ Medical Journal. In addition, some major journals have dedicated special forums for student participation such as Lancet Student and Student BMJ. Despite this rise in numbers, accessibility and citations, only a few of them manage to meet the quality standards for indexing in well-known academic databases, such as PubMed/Medline [2].

The impact and quality of these journals remains a source of debate. Student journals can help to improve undergraduate training by providing high-quality educational experience. Additionally, they can helpto motivate students to participate in research and to express their opinions and perspectives. The student-friendly peer-review system encourages students to publish their own research, promoting students with inexperience in publishing research to develop their style of writing. They also provide an opportunity for students to become accustomed to the submission and peer-review process. Moreover, exceptional editorial opportunities are offered for interested students to learn about medical journalism and to develop their critical appraisal skills providing trained editors for the future [3].

Notwithstanding these merits, student journals face major critiques and challenges. The peer-review system, which is managed mainly by undergraduates as editors and reviewers who lack enough clinical and critical appraisal skills to provide constructive reviews, may allow low-quality studies to reach publication resulting in a culture of weak studies. In addition, the lack of indexing and readability of these journals may deprive good articles from reaching their targeted audience and subsequent citations. Furthermore, the issues of sustainability in securing good-quality submissions, funds and dedicated editorial teams are other major concerns for the future of these journals [4].

To tackle these issues, it is important that these journals adopt a strict peer-review system in accordance with international editing guidelines, with experts leading the process. Additionally, funding agencies, academic institutions and professional publishing groups should collaborate with these journals to make sure that these efforts of future researchers are directed along the right way.

## Conflict of interest

Omar Aboshady is an associate editor of International Journal of Medical Students.

## Funding

None.
